# Analysis of the variation pattern in right upper pulmonary veins and establishment of simplified vein models for anatomical segmentectomy

**DOI:** 10.1007/s11748-016-0686-4

**Published:** 2016-07-19

**Authors:** Kimihiro Shimizu, Toshiteru Nagashima, Yoichi Ohtaki, Kai Obayashi, Seshiru Nakazawa, Mitsuhiro Kamiyoshihara, Hitoshi Igai, Izumi Takeyoshi, Akira Mogi, Hiroyuki Kuwano

**Affiliations:** 1 Division of General Thoracic Surgery, Integrative Center of General Surgery, Gunma University Hospital, 3-39-22 Showa-machi, Maebashi, Gunma 371-8511 Japan; 2 Department of Thoracic and Visceral Organ Surgery, Gunma University Graduate School of Medicine, 3-39-22 Showa-machi, Maebashi, Gunma 371-8511 Japan; 3 Department of General Thoracic Surgery, Maebashi Red Cross Hospital, 3-21-36 Asahi-cho, Maebashi, Gunma 371-0014 Japan

**Keywords:** Pulmonary vein, Three-dimensional computed tomography, Right upper lobe, Segmentectomy

## Abstract

**Objective:**

Thoracic surgeons must be erudite pulmonary vein variation when performing anatomical segmentectomy. We used three-dimensional CT (3DCT) to accumulate variations of the pulmonary veins of the right upper lobe (RUL) and created a simplified RUL vein model.

**Methods:**

We reviewed anatomical variations of the RUL pulmonary veins of 338 patients using 3DCT images, and classified them by position related with bronchus.

**Results:**

Of the “anterior” and “central” RUL veins, all could be classified into 4 types: 2 Anterior with Central types (Iab and Ib), 1 Anterior type, and 1 Central type. The Anterior with Central type was observed in 273 patients (81 %), and was further classified into two types according to the origin of the anterior vein. In the Iab type, the anterior vein originated from V1a to V1b (54 %) whereas, in the Ib type, the anterior vein originated from only V1b (26 %). The Central type, which had no anterior vein, was evident in 23 cases (7 %). These three types could be further divided into three subcategories by reference to the branching pattern of the central vein. The Anterior type, which had no central vein, was evident in 42 cases (12 %), and this type could be further categorized into two types, depending on the branching pattern of the anterior vein.

**Conclusion:**

We created a simplified RUL vein model to facilitate anatomical segmentectomy. Our models should find wide application, especially when thoracic surgery requiring anatomical RUL segmentectomy is planned.

**Electronic supplementary material:**

The online version of this article (doi:10.1007/s11748-016-0686-4) contains supplementary material, which is available to authorized users.

## Introduction

The need for anatomical pulmonary segmentectomy, which preserves more lung parenchyma volume than lobectomy does, is increasing [1–3]. However segmentectomy is technically more difficult than standard lobectomy because of the anatomical complexity of peripheral vessels and bronchi. Understanding pulmonary vein branches and their variation is especially important, because these veins are the boundaries of pulmonary segments and the optimal segmentectomy approach depends on the variation of the peripheral segmental pulmonary veins [4]. For segmentectomy in addition to demarcation line by air or blood current into the segment using indocyanine green, identification of segmental veins is essential [5]. In a previous study, we showed that branching of the right upper lobe (RUL) pulmonary vein could be classified into four types, whereas the more peripheral segmental veins branching patterns in those four types were more complex [6].

We have previously shown that 3DCT imaging is useful in assessing pulmonary vein anatomy prior to thoracic surgery [6, 7]. Oizumi et al. recently emphasized that 3DCT angiography was a powerful tool, enabling surgeons to identify intersegmental pulmonary veins and secure surgical margins when planning thoracoscopic lung segmentectomy [8]. However, even if preoperative 3DCT imaging is performed, it is difficult to understand pulmonary segmental anatomy without good understanding of pulmonary bronchovascular patterns, especially the branching patterns of pulmonary veins. Thus, anatomical models encompassing the variation of pulmonary veins, including peripheral segmental veins, have become increasingly important. Such models will allow general thoracic surgeons to plan safe and precise pulmonary segmentectomy. However, only a few systematic reports on RUL anatomy (including ours) have appeared [6, 9, 10]; these reports lack the detailed anatomical information that thoracic surgeons require prior to segmentectomy. The reports adequately describe variations in bronchovascular patterns, but do not carefully categorize the peripheral segmental veins. Also, the figures showing the segmental veins are too complex; they do not aid in an understanding of segmental anatomy.

The purpose of the present study was to explore relationships between the various types of pulmonary vein branching, and variations of peripheral segmental veins in the RUL, using data derived from 3D computed tomography (3DCT). We created simple anatomical vein models to aid thoracic surgeons who plan RUL segmentectomies.

## Patients and methods

### Reconstruction of 3DCT images

The method used to reconstruct 3DCT images has been previously described [6]. Briefly, the 3DCT bronchovascular patterns were analyzed using a 64-channel MDCT (SOMATOM Definition Flash; Siemens Healthcare, Berlin, Germany). Volume data from both the arterial and venous phases were transferred to a workstation running volume-rendering reconstruction software (Ziostation2; Ziosoft, Tokyo, Japan); the software converted the data to a 3DCT angiographic format. Radiology technicians processed all 3D images and thoracic surgeons confirmed the validity of all reconstructions.

### Patient preparation and examination

Between January 2010 and August 2015, all patients with pulmonary and mediastinal lesions underwent 3DCT prior to surgery. The 338 most recent consecutive cases (192 males, 146 females; mean age 65 years) were analyzed. Patient characteristics are summarized in e-Table 1. The segmental and subsegmental veins were named by reference to their relationships with the segmental artery and bronchi. We used 3DCT to analyze the bronchovascular pattern of the RUL. Data analysis was independently performed by three thoracic surgeons (K.S., T.N., and Y.O.). If different views were expressed, a final decision was made after discussion.

This retrospective study was approved by the Institutional Review Board for Clinical Trials of Gunma University Hospital.

### Definitions of the pulmonary and segmental veins

The nomenclature used to describe the pulmonary segmental veins is that of Nagashima, Yamashita, and Boyden (e-Table 2) [6, 9, 10]. Briefly, branching of the pulmonary vein was defined as follows (1) anterior vein (V. ant): The anterior vein originates from V1b and descends down the anterior side of the upper lobe bronchus, finally draining into the superior pulmonary vein from the mediastinal side; (2) central vein (V. cent): the central vein originates from V2a and descends through the center of the upper lobe, between B2 and B3, finally draining into the superior pulmonary vein from the interlobar side. Branching of the RUL pulmonary veins was divided into three types in terms of function: the intersegmental veins (V^1^b, V^2^a, V^2^c) run between segments, the intersubsegmental veins (V^1^a, V^2^b, V^3^a) run between subsegments, and the surface veins (V^2^t, V^3^b, V^3^c) run along the surface, or within fissures of the RUL (e-Table 2). Basically, V^1^ drains into V. ant, and V^2^ into V. cent or V^2^t. Thus, when V^1^ drains into V. cent and V^2^ drains into V. ant, the intersegmental or intersubsegmental veins are termed VX (VX^1^a, VX^1^b, VX^2^a, VX^2^c). VX^2^a was further subclassified into two patterns by reference to anatomical position; VX^2^a runs through the center of the RUL between B^1^ and B^3^ and VXX^2^a runs on the mediastinal surface of S^1^ (e-Table 2; Fig. 5d).

## Results

### Branching of the pulmonary vein

**Fig. 1:**
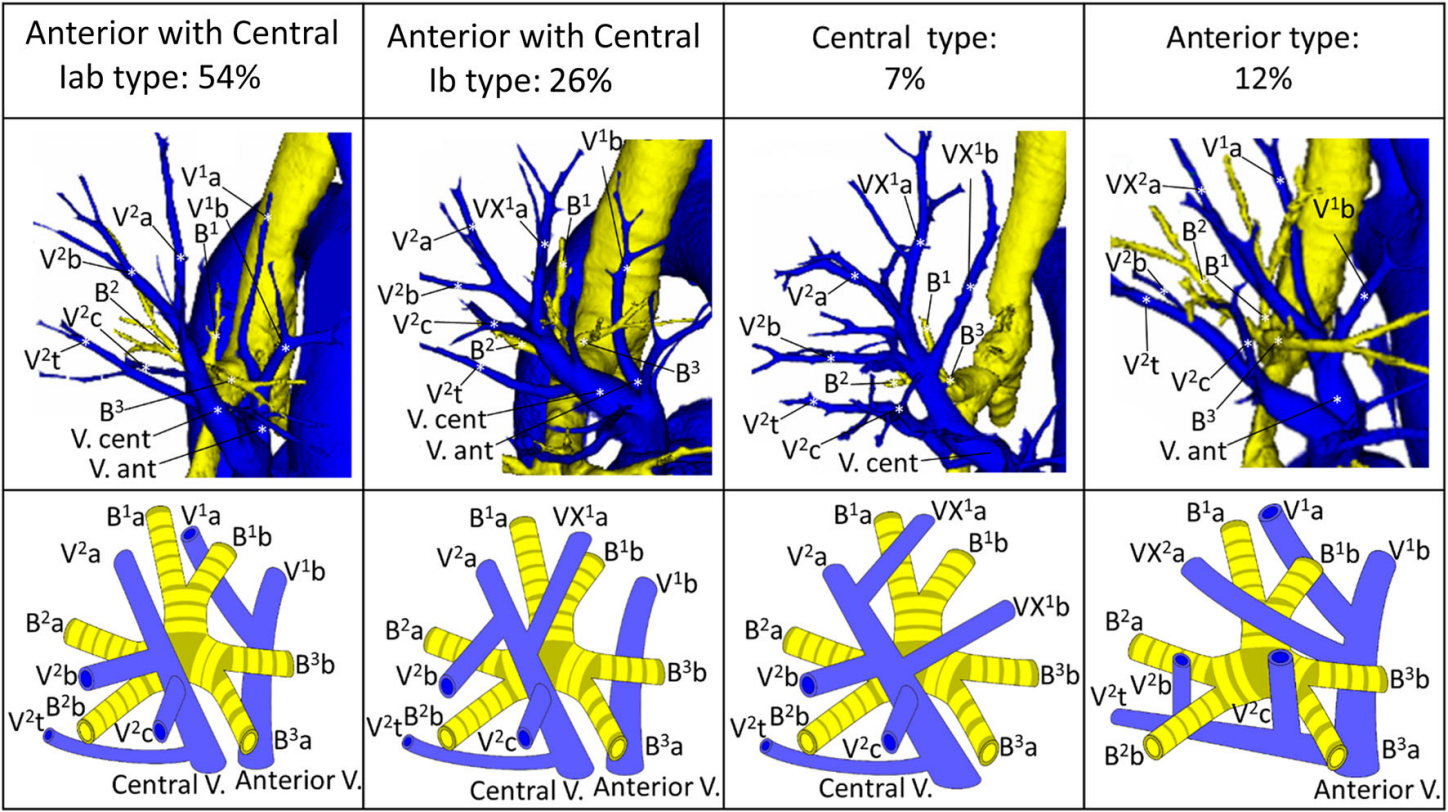
Types of branching of right upper lobe veins and each simplified models

**Fig. 2:**
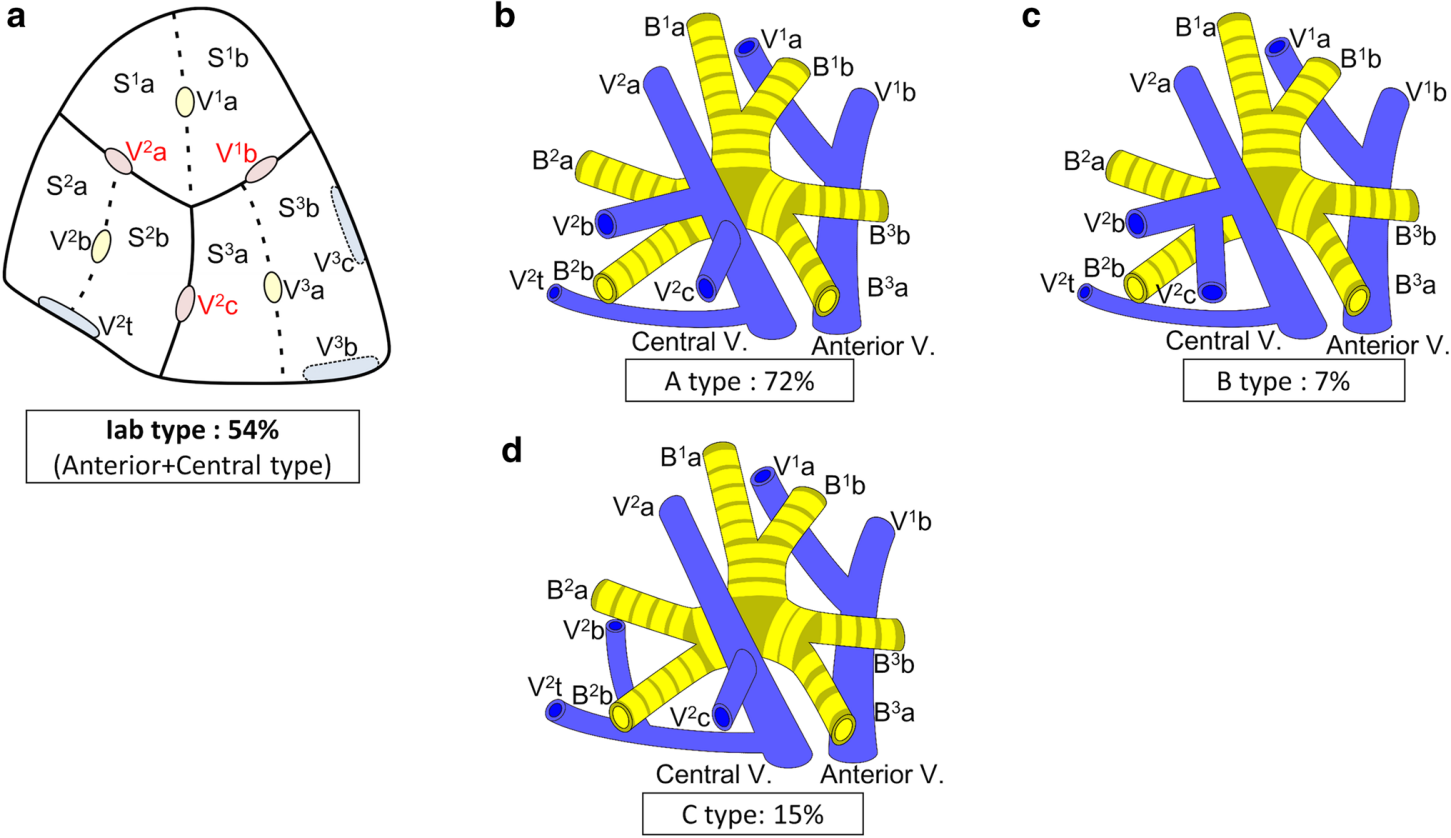
Simplified models of the Iab type (Anterior with Central type)

**Fig. 3:**
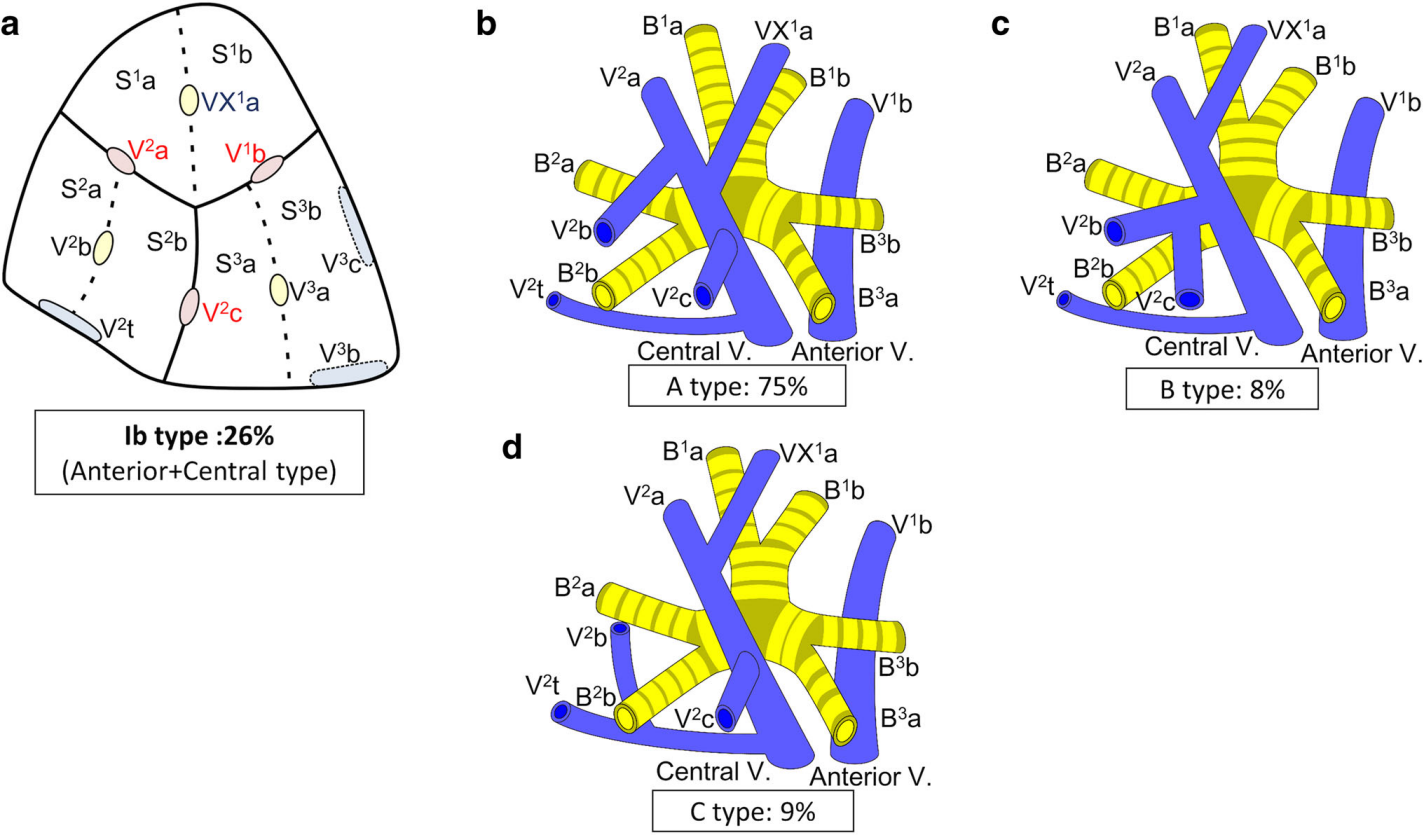
Simplified models of the Ib type (Anterior with Central type)

**Fig. 4:**
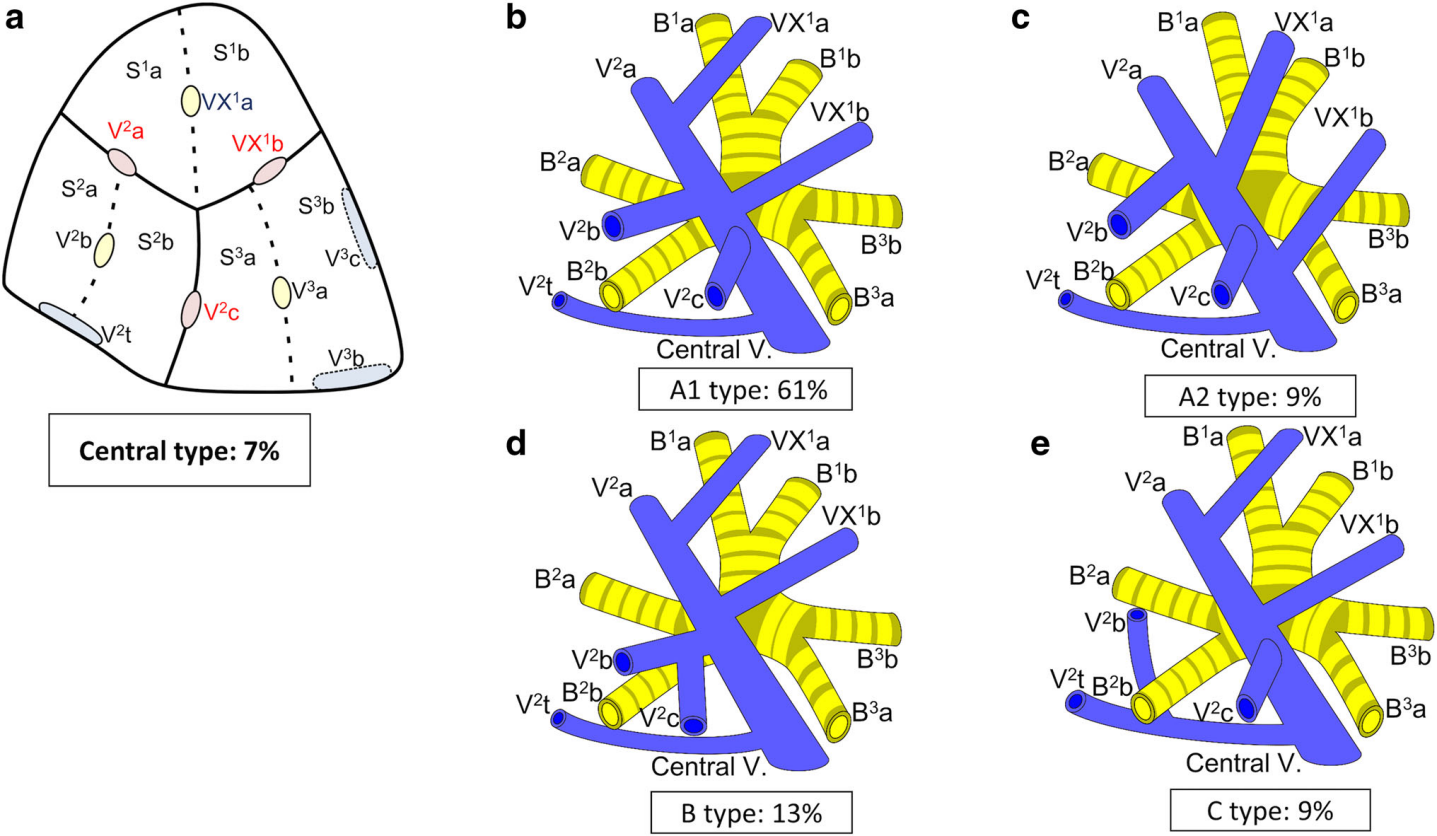
Simplified models of the Central type

**Fig. 5:**
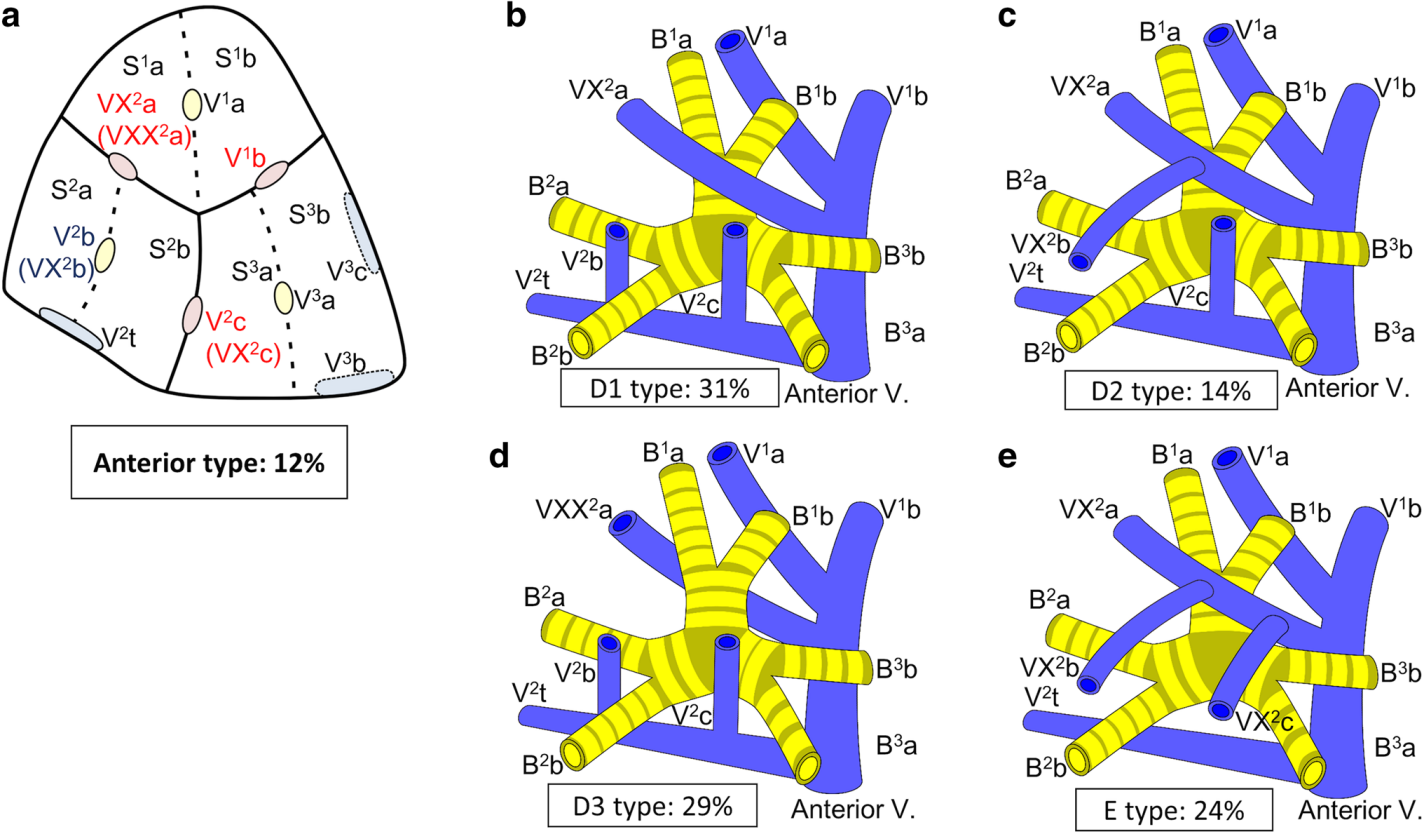
Simplified models of the Anterior type

Branching of the pulmonary vein was classified into four types. The “Anterior with Central” form was evident in 273 cases (81 %), and was further classified into two types (Iab and Ib) (Fig. 1). In the Iab type, V. ant originates from V1a and V1b; this variation was present in 184 cases (54 %) (Figs. 1, 2). In the Ib type, V. ant originates only from V1b, whereas V^1^a is termed VX^1^a and drains into V. cent. This variation was seen in 89 cases (26 %) (Figs. 1, 3). The Central type, in which V^1–2^ drains into V. cent, was seen in 23 cases (7 %) (Figs. 1, 4). Each of these three types (Iab, Ib, and Central) could be further divided into three anatomical categories (A, B, and C) depending on the branching pattern of the central vein. The A type is independent; V^2^b and V^2^c each drain independently into V^2^a (Figs. 2b, 3b, 4b, c). In the B type, V^2^b and V^2^c share a common trunk; this drains into V^2^a (Figs. 2c, 3c, 4d). In the C type, V^2^t and V^2^b share a common trunk; this drains into V^2^a at a location central to V^2^c (Figs. 2d, 3d, 4e). The Anterior type, in which V^1–2^ drains into V. ant, was evident in 42 cases (12 %) (Figs. 1, 5), and this type could also be divided into two categories depending on the branching pattern of the anterior vein. These were the D type (V^2^t and V^2^c share a common trunk; VX^2^a drains into V. ant and V^2^c into V^2^t) (Fig. 5b–d) and the E type (VX^2^a, VX^2^b, and VX^2^c share a common trunk; this drains into V. ant) (Fig. 5e). All combinations of pulmonary vein branching types and the anatomical classifications of the peripheral branches are listed in Table 1. We further analyzed the V^3^ drainage patterns of the RUL. However, variations were numerous and defied categorization (e-Table 3). Furthermore, with the exception of V^3^a, V^3^ is not anatomically involved in pulmonary segmental structure. Thus, to avoid confusion, the V^3^ veins were excluded from the simplified models.

**Table 1:** The intersegmental and intersubsegmental pulmonary vein patterns of each of the four branching types

Pulmonary vein type	Central and Anterior vein pattern	Anatomical classification	Type	*N*	%	Fig. no
Iab type (Anterior with Central type), *n* = 184 (54 %)	V^2^a, V^2^b, V^2^c	Independent type	A	132	72	Figures 1, 2b
	V^2^a, V^2^b + V^2^c	V^2^b and V^2^c common trunk type	B	13	7	Figure 2c
	V^2^a, V^2^c, V^2^t + V^2^b	V^2^t and V^2^b common trunk type	C	28	15	Figure 2d
	Aberrant V^2^	Other	Minor	5	3	e-Figure 1a
	No V^2^a			3	2	e-Figure 1b
	V^2^a from V^2^t			3	2	e-Figure 1c
Ib type (Anterior with Central type), *n* = 89 (26 %)	V^2^a, (V^2^b, VX^1^a or VX^1^a, V^2^b), V^2^c	Independent type	A	67	75	Figures 1, 3b
	V^2^a, VX^1^a, V^2^b + V^2^c	V^2^b and V^2^c common trunk type	B	7	8	Figure 3c
	V^2^a, VX^1^a, V2c, V^2^t + V^2^b	V^2^t and V^2^b common trunk type	C	8	9	Figure 3d
	Aberrant V^2^	Other	Minor	2	2	
	V^2^a from V^2^t			3	3	
	V^2^c from V^2^t			2	2	
Central type, *n* = 23 (7 %)	V^2^a, (VX^1^a, V^2^b, VX^1^b)^a^, V^2^c	Independent type	A1	14	61	Figures 1, 4b
	V^2^a, (V^2^b, VX^1^a or VX^1^a, V^2^b), V^2^c, VX^1^b		A2	2	9	Figure 4c
	V^2^a, VX^1^a, VX^1^b, V^2^b + V^2^c	V^2^b and V^2^c common trunk type	B	3	13	Figure 4d
	V^2^b from V^2^t	V^2^t and V^2^b common trunk type	C	2	9	Figure 4e
	V^2^a from V^2^t	Other	Minor	2	9	
Anterior type, *n* = 42 (12 %)	VX^2^a, V^2^t + V^2^b + V^2^c	V^2^t and V^2^c common trunk type	D1	13	31	Figures 1, 5b
	VX^2^a + VX^2^b, V^2^t + V^2^c		D2	6	14	Figure 5c
	VXX^2^a, V^2^t + V^2^b + V^2^c		D3	12	29	Figure 5d
	VX^2^a + VX^2^b + VX^2^c, V^2^t	VX^2^a, VX2b and VX^2^c common trunk type	E	10	24	Figure 5e
	VXX^2^a, VX^2^b, VX^2^c, V^2^t	Other	Minor	1	2	

^a^Random order

### The intersegmental and intersubsegmental pulmonary vein patterns of each of the four types of pulmonary vein branching patterns

#### Iab type

Iab was the most common form of branching (Fig. 1). Iab was classified into three types by reference to V. cent (as described above). The A type was present in 132 cases (72 %) (Fig. 2b; Table 1), the B type in 13 cases (7 %) (Fig. 2c; Table 1), and the C type in 28 cases (15 %) (Fig. 2d; Table 1). Three anomalous V^2^ drainage patterns were recognized: an “Aberrant V^2^ type”, in which V^2^ drained into the inferior pulmonary vein, crossing behind the intermediate bronchus (5 cases: 3 %) (e-Fig. 1a; Table 1), a “no V^2^a type”, which lacked V^2^a (3 cases: 2 %) (e-Fig. 1b; Table 1), and a “V^2^a from V^2^t type’’, in which V^2^a drained into V^2^t (3 cases: 2 %) (e-Fig. 1c; Table 1).

#### Ib type

Ib was the second most common form of branching (Fig. 1). In Ib, V. ant originates from only V^1^b, whereas VX^1^a draining into V. cent. Ib was classified into three types by reference to the V. cent pattern, as for the Iab type (described above): the A type was present in 67 cases (75 %) (Fig. 3b; Table 1), the B type in 7 (8 %) (Fig. 3c; Table 1), and the C type in 8 (9 %) (Fig. 3d; Table 1). Three anomalous V^2^ drainage patterns were observed: the “Aberrant V^2^ type” (2 cases: 2 %) (Table 1), the “V^2^a from V^2^t type” (3 cases: 3 %) (Table 1), and the “V^2^c from V^2^t type”, in which V^2^c drained into V^2^t (2 cases: 2 %) (Table 1).

#### Central type

The Central form, in which V^1–2^ drains into V. cent, was seen in 23 cases (7 %) (Fig. 1). “Central” was first classified into three types, as for Iab and Ib (described above). However, the A type was further subclassified into two subtypes by reference to the branching site of VX^1^b, which is a surgically important intersegmental vein that separates S1 from S3. In the A1 subtype, VX^1^b drains into V^2^a at the peripheral side of V^2^c (14 cases: 61 %) (Fig. 4b; Table 1). On the other hand, in the A2 type, VX^1^b drains into V2a at the central side of V^2^c (2 cases: 9 %) (Fig. 4c; Table 1). The B type was present in 3 cases (13 %) (Fig. 4c; Table 1). The C type was present in 2 cases (9 %) (Fig. 4d; Table 1).

#### Anterior type

The Anterior form, in which V. cent is absent and V^1–2^ drains into V. ant and V^2^t, was present in 42 cases (12 %) (Fig. 1). V. ant was classified into two types: the D type was present in 32 cases (76 %) (Fig. 5b–d) and the E type in 10 (24 %) (Fig. 5e). Furthermore, the D type was subclassified into three subtypes. D1, in which VX^2^a drains into V^1^b, and V^2^b and V^2^c drains into V^2^t, was present in 13 cases (31 %) (Fig. 5b). D2, in which the common trunk of VX^2^a + VX^2^b drains into V^1^b, and V^2^c drains into V^2^t, was present in 6 cases (14 %) (Fig. 5c). In the D3 subtype, VXX^2^a runs along the mediastinal surface of S^1^ and drains into V. ant, and V^2^b and V^2^c drain into V^2^t. This subtype was present in 12 cases (29 %) (Fig. 5d).

## Discussion

We previously used 3DCT to describe the right upper pulmonary bronchovascular patterns, and the frequencies of variations therein [6, 7]. In the present study, we used 3DCT to further analyze branching of the RUL pulmonary veins, focusing on variations among the peripheral segmental veins, in 338 patients. The frequency of each branching type was similar to that noted in our previous report [6]. However, in our previous work, we did not analyze variations in peripheral segmental veins, and thus could not categorize these segmental vessels in terms of branching type. Accurate preoperative data on peripheral pulmonary segmental veins is essential when segmentectomy is planned. We re-analyzed our 3DCT bronchovascular database and created simplified models focusing particularly on the surgically important segmental veins.

For example, V^2^c, which is an intersegmental vein lying between S^2^ and S^3^, drains into V. cent in the “Anterior with Central” and “Central” types (Figs. 1, 2, 3, 4). Thus, when S^2^ or S^3^ segmentectomy is planned for such patients, V. cent must be dissected in a center-to-periphery direction to identify V^2^c. In contrast, in the Anterior type, V^2^c drains into V^2^t or V. ant. It is therefore necessary to dissect V^2^t or V. ant, but not V. cent, to identify V^2^c (Fig. 5). Furthermore, when S^1^ or S^2^ segmentectomy is planned for an Anterior type, if V^2^c is erroneously identified as V. cent, it is impossible to identify V^2^a (in reality, VX^2^a or VXX^2^a) because V^2^a drains into V. ant. However, if 3DCT imaging and our simplified models are used to preoperatively explore branching in the pulmonary veins and the type of peripheral intersegmental and intersubsegmental veins of the RUL, anatomical segmentectomy can be accurately performed. Similarly, V^1^b, which is an intersegmental vein lying between S^1^ and S^3^, drains into V. ant in the “Anterior with Central” and “Anterior” types (Figs. 1, 2, 3, 5). Thus, when S^1^ or S^3^ segmentectomy is planned, it is necessary to dissect V. ant in a center-to-periphery direction to identify V^1^b. In contrast, in the Central type, VX^1^b drains into V. cent, so V. cent must be dissected to identify VX^1^b (Fig. 4). Furthermore, VX^1^b branching exhibits at least two patterns in the Central type; in one, VX^1^b drains into V. cent at the peripheral side of V^2^c (A1 type) (Fig. 4b), and in the other, VX^1^b drains into V. cent at the central side of V^2^c (A2 type) (Fig. 4c). As V^2^c is an important intersegmental vein, especially in the context of S1 and S2 segmentectomy, preoperative recognition of the anatomical relationship between V^2^c and VX^1^b in the Central type is necessary. Thus, we further classified the A subtype of the Central type into A1 and A2, depending on the VX^1^b branching pattern. For the reasons articulated above, preoperative recognition of the vein branching type, and tactical preparation using our simplified models, is critical to ensure anatomically accurate RUL segmentectomy.

Our work has several limitations. First, we did not integrate the segmental artery or the bronchi into our models. Variations in segmental veins are very closely associated with those of the segmental bronchi and arteries [6]. However, we focused principally on the relationships between pulmonary veins and the lung segments because the models would become very complex if we included variations in the segmental bronchi and arteries. Second, we used 3DCT data exclusively; it is possible that these may vary somewhat from real anatomical findings.

## Conclusion

This is the first report to categorize the intersegmental and intersubsegmental pulmonary vein patterns of the RUL, and to create simplified models for use when planning anatomical segmentectomy. We believe that our pulmonary vein data, our new nomenclature, and our simplified models will be of assistance in both preoperative simulation and intraoperative navigation when anatomical RUL segmentectomy is planned and underway.

## Electronic supplementary material

Below is the link to the electronic supplementary material.

Supplementary material 1 (DOCX 19 kb)

Supplementary material 2 (TIFF 2030 kb)
