# Supplementary material for: Analysis of the variation pattern in right upper pulmonary veins and establishment of simplified vein models for anatomical segmentectomy
Source: Gen Thorac Cardiovasc Surg. 2016 Jul 19;64(10):604–11. doi: 10.1007/s11748-016-0686-4 (PMC5035324; doi:10.1007/s11748-016-0686-4)
Supplement: Supplementary file 1 — Supplementary material 1 (DOCX 19 kb) [file 11748_2016_686_MOESM1_ESM.docx]

**Supplementary Figure 1 (e-Figure 1)**. Minor branching patterns of the segmental veins in Iab type.

e-Table 1: Patient Characteristics

|  | No. of Patients | % |
| --- | --- | --- |
| Age, year |  |  |
| Median | 67 | |
| Range | (15-91) | |
| Gender |  |  |
| Male | 192 | 57 |
| Female | 146 | 43 |
| Disease |  |  |
| Primary lung cancer | 231 | 68 |
| Mediastinal tumor | 37 | 11 |
| Metastatic lung cancer | 30 | 9 |
| Others | 40 | 12 |

e-Table 2: Nomenclature of the RUL veins.

|  | | Position | Function | Drainage vein | Pulmonary vein type |
| --- | --- | --- | --- | --- | --- |
| V^1^ ( V. apicalis ) | V^1^a | between S^1^a and S^1^b | intersubsegmental vein | Anterior vein | Iab, Anterior |
|  | VX^1^a |  |  | Central vein | Ib, Central |
|  | V^1^b | between S^1^and S^3^ | intersegmental vein | Anterior vein | Iab, Ib, Anterior |
|  | VX^1^b |  |  | Central vein | Central |
| V^2^ ( V.dorsalis ) | V^2^a | between S^1^and S^2^ | intersegmental vein | Central vein | Iab, Ib, Central |
|  | VX^2^a |  |  | Anterior vein, between S^1^ and S^3^ | Anterior |
|  | VXX^2^a |  |  | Anterior vein, mediastinal surface of S^1^ |  |
|  | V^2^b | between S^2^a and S^2^b | intersubsegmental vein | Central vein or V^2^t | Iab, Ib, Central, Anterior |
|  | VX^2^b |  |  | Anterior vein | Anterior |
|  | V^2^c | between S^2^and S^3^ | intersegmental vein | Central vein or V^2^t | Iab, Ib, Central, Anterior |
|  | VX^2^c |  |  | Anterior vein, VX^2^a or VXX^2^a common trunk | Anterior |
|  | V^2^t | between S^2^and S^6^ | surface vein | Anterior vein or Central vein | Iab, Ib, Anterior, Central |
| V^3^ ( V. ventralis ) | V^3^a | between S^3^a and S^3^b | intersubsegmental vein | Anterior vein or Central vein or SPV* | Iab, Ib, Anterior, Central |
|  | V^3^b | below S^3^b | surface vein | Anterior vein or Central vein or SPV* | Iab, Ib, Anterior,Central |
|  | V^3^c | mediastinal side of S^3^b | surface vein | Anterior vein or Central vein or SPV* |  |
| *SPV: superior pulmonary vein | | |  |  |  |

e-Table 3: V3 drainage patterns of the RUL

| V^3^ | Iab | | Ib | | Central | | Anterior | |
| --- | --- | --- | --- | --- | --- | --- | --- | --- |
|  | n | % | n | % | n | % | n | % |
| V^3^a and V^3^b comon trunk → SPV, V^3^c → V. ant | 12 | 6.5 | 18 | 20.2 | 0 | 0.0 | 2 | 4.8 |
| V^3^a and V^3^b comon trunk and V3c → SPV | 13 | 7.1 | 8 | 9.0 | 0 | 0.0 | 0 | 0.0 |
| V^3^a → V.cent, V^3^b and V^3^c common trunk → SPV | 14 | 7.6 | 6 | 6.7 | 0 | 0.0 | 1 | 2.4 |
| V^3^a → V.cent, V^3^b → SPV, V^3^c → V. ant | 11 | 6.0 | 7 | 7.9 | 0 | 0.0 | 2 | 4.8 |
| V^3^a → V.cent, V^3^c → V. ant, V^3^b does not exist | 12 | 6.5 | 3 | 3.4 | 0 | 0.0 | 0 | 0.0 |
| V^3^a and V^3^b comon trunk → SPV, V^3^c does not exist | 8 | 4.3 | 3 | 3.4 | 4 | 17.4 | 0 | 0.0 |
| V^3^a and V^3^b comon trunk → V. cent, V^3^c → V.ant | 12 | 6.5 | 1 | 1.1 | 0 | 0.0 | 0 | 0.0 |
| others |  | 0.0 | 43 | 48.3 | 19 | 82.6 | 37 | 88.1 |
| total | 184 | 100.0 | 89 | 100.0 | 23 | 100.0 | 42 | 100.0 |

V. ant: anterior vein, V. cent: central vein, SPV: superior pulmonary vein
