# Supplementary figures and images for: Analysis of the variation pattern in right upper pulmonary veins and establishment of simplified vein models for anatomical segmentectomy
Source: Gen Thorac Cardiovasc Surg. 2016 Jul 19;64(10):604–11. doi: 10.1007/s11748-016-0686-4 (PMC5035324; doi:10.1007/s11748-016-0686-4)

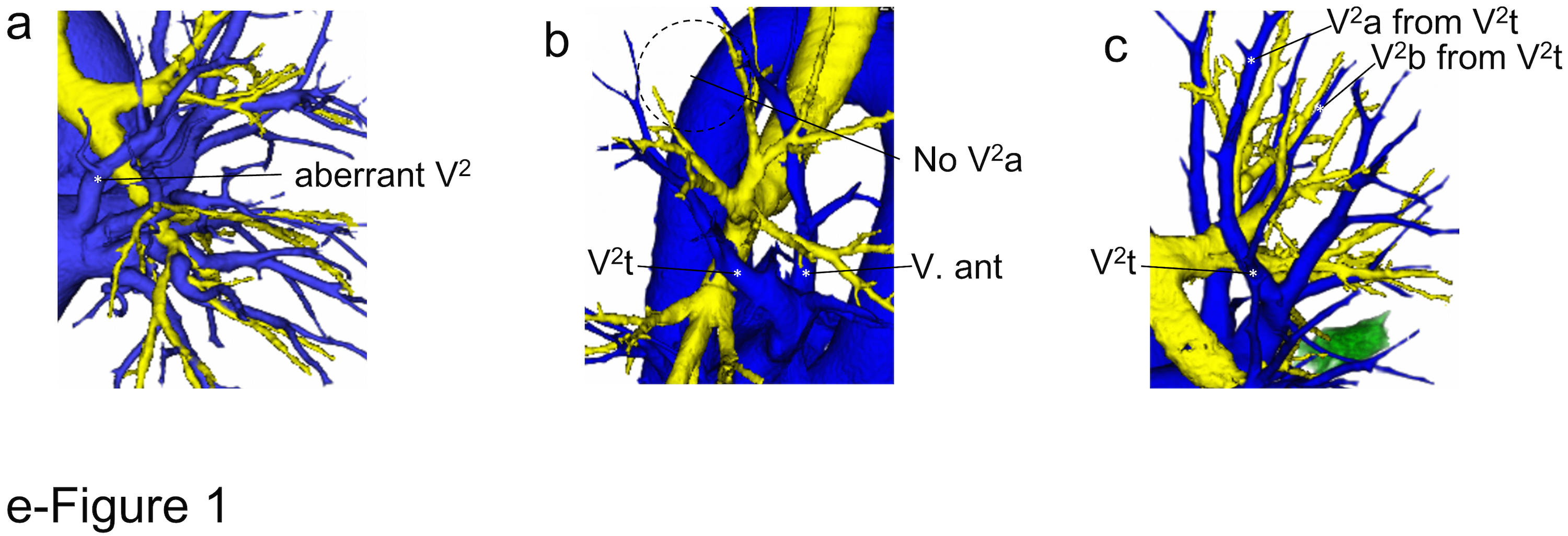

Supplement: Supplementary file 2 — Supplementary material 2 (TIFF 2030 kb) [file 11748_2016_686_MOESM2_ESM.tiff]
